# Risk factors of venous thrombosis in knee joint endoprosthesis

**DOI:** 10.1186/cc11024

**Published:** 2012-03-20

**Authors:** SV Vlasov, IV Vlasova

**Affiliations:** 1 Scientific Clinical Center of Miner's Health Protection, Leninsk-Kuznetsky, Russia

## Introduction

The high risk of thromboembolic complications after knee joint prosthetics is conditioned by the series of surgical intervention particularities. The influence of intraoperative tourniquet usage on the leg deep venous thrombosis frequency was studied.

## Methods

The study included 125 patients with gonarthrosis of degree III who received total knee joint endoprosthesis. There were 26 men and 99 women at the age of 36 to 77 (60.7 ± 8.03). For all patients, spinal anesthesia in combination with long-term epidural blockade for postsurgical pain relief was performed. The antithrombotic measures included Klexan 40 mg, 12 to 15 hours before surgery and 8 hours after it. Color mapping of the lower leg vessels with an Acuson 128XP/10c scanner was performed before surgery, on 4 to 5 days after prosthetics and before discharge from the in-patient department. In addition, all patients underwent study of endothelial vasodilating function using the method proposed by Celermajer and colleagues [1].

## Results

On 4 to 5 days after surgery, leg deep venous thrombosis was found in 11 patients (8.8% of all patients after prosthetics). For decrease of intraoperative blood loss the tourniquet was applied onto the middle third of the leg in 77 patients (60.6%). In this group DVT was found in 10.4% of cases. In the nontourniquet group (48 patients) DVT was found in 6.25%. The differences in the complication frequency were not statistically valid. The data from duplex scanning showed that 43 patients (34.4%) before surgery had changes in the lower leg veins in view of varicose subcutaneous veins and post-thrombophlebitic syndrome combined with disorders of endothelial vasodilating function and low venous tone. Tourniquet use in patients with venous pathology resulted in DVT in 30% (five of 15 patients). When a tourniquet was not used in patients with venous disease, DVT was found only in one of 28 patients (3.5%). The test showed a significant difference in the frequency of thromboembolic complications in these groups (*P *< 0.001).

## Conclusion

Therefore, using a tourniquet in patients with evident base venous pathology in terms of varicose subcutaneous veins or post-thrombophlebitic syndrome in total knee joint endoprosthetics is a risk factor for venous thrombosis development.
